# DPP6 Loss Causes Age-Dependent Sleep Dysregulation and Depression-like Phenotypes Linked to Neurodegeneration

**DOI:** 10.3390/ijms27073224

**Published:** 2026-04-02

**Authors:** Lin Lin, Ashley E. Pratt, Dax A. Hoffman

**Affiliations:** Molecular Neurophysiology and Biophysics Section, Program in Developmental Neuroscience, *Eunice Kennedy Shriver* National Institute of Child Health and Human Development, National Institutes of Health, Bethesda, MD 20892, USA; linl2@mail.nih.gov (L.L.); ashley.pratt@nih.gov (A.E.P.)

**Keywords:** DPP6, sleep disorders, REM, NREM, depression, body temperature, Alzheimer’s disease, dementia, neurodegeneration

## Abstract

Sleep disturbances are early hallmarks of Alzheimer’s disease (AD) and other dementias, yet the molecular mechanisms remain poorly understood. We previously showed that dipeptidyl aminopeptidase-like protein 6-knockout (DPP6-KO) mice exhibit accelerated neurodegeneration with synaptic loss, neuronal death, and circadian dysfunction resembling AD pathology. Here, we investigate whether DPP6 deficiency directly causes sleep dysregulation and assess age-dependent effects using wireless EEG/EMG telemetry, behavioral monitoring, and body temperature recordings. We found striking age-dependent sleep phenotypes in DPP6-KO mice. Adult (3-month) DPP6-KO mice showed hyperactivity-driven REM sleep increases, while aged (12-month) DPP6-KO mice developed insomnia with fragmented sleep architecture. Critically, aged DPP6-KO mice exhibited decreased REM latency, a biomarker of depression, which we confirmed by behavioral assays. Conversely, DPP6 overexpression in aged wild-type mice increased NREM duration and reduced sleep fragmentation, demonstrating a protective effect. Throughout aging, DPP6-KO mice showed dysregulated locomotor activity and body temperature rhythms, suggesting broader disruption of circadian and metabolic homeostasis. These findings establish DPP6 as a critical regulator of sleep architecture whose loss recapitulates key sleep disturbances observed in AD/dementia. The progressive nature of sleep dysfunction in DPP6-KO mice, from REM abnormalities to insomnia, parallels human disease progression and positions DPP6 as a potential therapeutic target for sleep-related symptoms in neurodegenerative disorders.

## 1. Introduction

Sleep is an essential process that is evolutionarily conserved across many species. Although our understanding of sleep’s functional role is incomplete, there is mounting evidence that it promotes a healthy immune system, facilitates the consolidation of memories, and reduces the risk of mental health disorders [1,2,3]. Two thoroughly characterized stages of sleep, slow-wave (SWS) and rapid eye movement (REM) sleep, have both been implicated in hippocampal-dependent memory [2,4]. SWS is a stage of deep sleep that facilitates post-learning replay and memory consolidation, while REM sleep consists of increased neurological activity, dreaming, and synaptic pruning that seems to promote memory consolidation [2,4].

Dysfunctional sleep has also been reported as a common comorbidity with multiple neurological disorders, including autism spectrum disorder, depression, and neurodegenerative diseases [5,6,7,8]. Time spent in SWS and REM sleep has been shown to decline with age, with more pronounced effects in SWS that may be tied to age-related cognitive decline [9]. Although dysregulated sleep is clearly implicated in altered neurological function, it is difficult to disentangle the extent to which abnormal sleep contributes to, or results from, the symptoms associated with these conditions. Greater characterization of disordered sleep and its underlying processes may reveal how sleep contributes to broader neurological function, especially in disease states.

Alzheimer’s disease (AD) is a disorder of particular concern, as it is estimated to affect ~6 million people in the United States, with projections to increase to ~13 million by 2060 [10]. AD is characterized by significant cognitive and memory impairments that exceed symptoms of normal aging. Additional symptoms of AD include anxiety, depression, and the accumulation of molecular markers such as amyloid plaque and tau protein in the brain. Over 25% of patients diagnosed with AD are reported to exhibit some form of sleep disturbance, characterized by highly fragmented sleep, daytime sleepiness, altered body temperature regulation, and increased latency to sleep [7,8,11]. Reduced SWS is also more pronounced in AD patients in comparison to healthy-aging controls [9]. These phenotypes can emerge early in the behavioral onset of AD-related symptoms and increase with disease progression, possibly exacerbating the disease condition [7,12]. Given the prevalence of sleep disturbances among AD patients, it is valuable to explore the physiological processes that underlie this phenotype, which may elucidate processes involved in AD progression and reveal potential therapeutic targets. Investigation of these phenotypes may also reveal the processes that drive common comorbidities, such as depression, which also has a higher prevalence among AD patients [13].

Previous work has identified a link between AD-like pathology and the dipeptidyl aminopeptidase-like protein 6 (DPP6). DPP6 is a transmembrane protein that serves as an auxiliary subunit for the voltage-gated potassium channel Kv4.2 and regulates hippocampal excitability and plasticity through this interaction [14,15]. DPP6 has also been shown to work independently from Kv4.2 to promote filopodia growth and stability, as well as spine formation and dendritic arborization [16]. Impaired DPP6 expression in mice (DPP6-KO) causes phenotypes such as increased amyloid-β and hyper-phosphorylated tau, reduced brain volume, and deficits in learning and memory, which mirrors the pathology observed in patients with AD [17]. The identification of a novel NeuN+ structure in DPP6-KO mice also points to a connection between DPP6 and AD, as these puncta colocalize with markers of AD and are more abundant in aging and AD mouse models [18,19]. These findings suggest that in addition to DPP6’s involvement in neurodevelopmental processes, it may also serve a neuroprotective role against symptoms associated with aging and AD.

Case studies of patients with DPPX–antibody-associated encephalitis, a rare condition in which autoimmunity targets the DPP6 protein, have also revealed phenotypes such as cognitive impairments, depression and anxiety [20,21]. Furthermore, 41.3% of these patients exhibit significant sleep disturbances such as insomnia or hypersomnia [22]. In alignment with these findings, we recently reported that aging (12 month-old) DPP6-KO mice show altered locomotor patterns across a 24 h period that may indicate abnormal activity and circadian rhythm [19]. In comparison to wild-type (WT), these mice exhibit hyperactivity in ambulation and fine movements during the light-on period. These results suggest that DPP6 might contribute to the regulation of the sleep–Wake cycle, but more direct evidence is required to determine whether sleep is altered in these mice. To further investigate this phenotype and directly measure sleep across experimental groups, we measured sleep in the present study with the use of an implanted wireless telemetry device to record electroencephalogram (EEG), electromyography (EMG), body temperature, and locomotion data in vivo.

Data collected for 2 days over a 24 h period revealed that aging (12 mo) DPP6-KO mice exhibit significantly reduced and highly fragmented sleep, as well as reduced non-REM (NREM) and increased REM sleep durations. Aging DPP6-KO mice show depressive behaviors with an increase in immobility shown by the tail suspension test, and decreased preference of 1% sucrose solution in the sucrose preference task. Adult (3 mo) DPP6-KO mice also exhibit mild sleep phenotypes, primarily in increased REM sleep time, as well as increased locomotion; however, we did not observe significant depressive behaviors in adult DPP6-KO mice. We also show that overexpression of DPP6 in 12 mo WT mice increases NREM sleep time and reduces fragmented sleep, supporting the hypothesis that DPP6 may promote healthy sleep during aging. Finally, our study also revealed altered body temperature in DPP6-KO mice in comparison to WT, suggesting differences in metabolic rate across groups. Altogether, these results provide novel evidence that DPP6 expression contributes to the regulation of sleep and metabolic function in mice.

## 2. Results

### 2.1. Aging DPP6-KO Mice Show Insomnia and Fragmented Sleep

**Figure 1:**
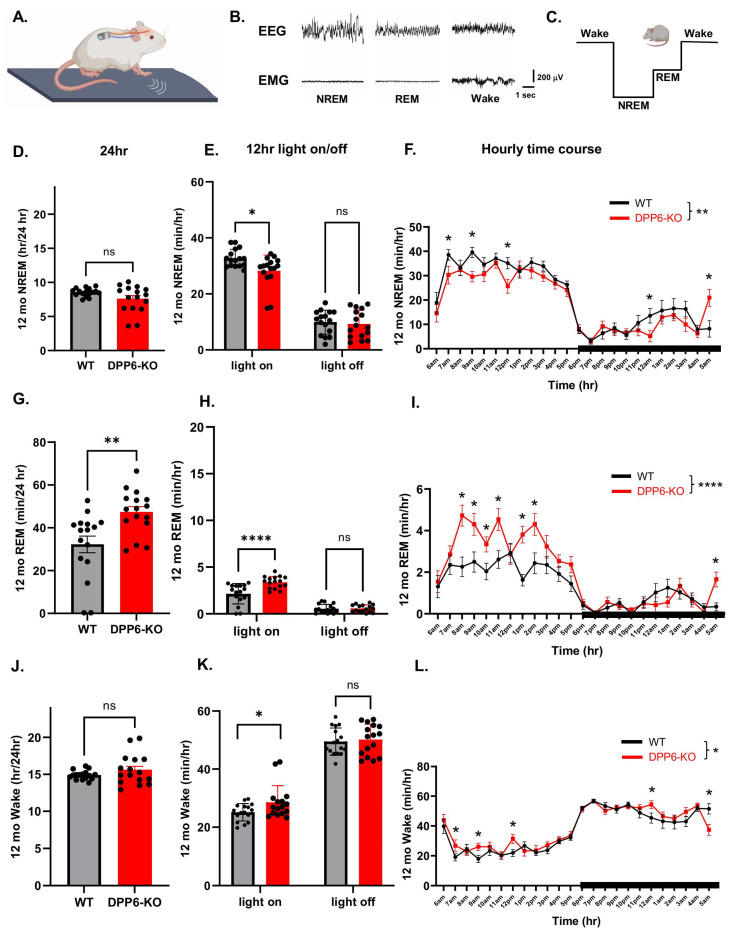
**Aging DPP6-KO mice have insomnia with a significant decrease in NREM sleep.** (**A**) Illustration showing the DSI implant wireless recording system. Mice were housed under a 12 h light/dark cycle with the lights on at 6 AM and lights off at 6 PM. (**B**) Representative EEG and EMG traces in NREM, REM and Wake stages. (**C**) Normal mouse sleep cycle. (**D**–**F**): Aging DPP6-KO mice have significantly less total NREM sleep during the 12 h light-on sleep cycle ((**E**), n = 8 mice each, two-way ANOVA, * *p* < 0.05) and hourly time course for 24 h (**F**) compared to WT. Two-way ANOVA indicated a main effect of genotype on NREM sleep (F (1, 720) = 10.38; ** *p* < 0.01, (**F**)). Furthermore, we found an interaction effect between genotype and time on NREM sleep (F (23, 720) = 1.659; * *p* < 0.05, (**F**)), and Tukey post hoc analysis showed a decrease in NREM sleep of 12 mo DPP6-KO mice at 7AM, 9AM, 12PM and 12AM (* Tukey’s test: *p* < 0.05, (**F**)). But there was no significant decrease in 12 h light-off ((**E**), two-way ANOVA, *p* > 0.05) and total 24 h ((**D**), unpaired *t*-tests, *p* > 0.05). ((**G**–**I**): Increased total REM sleep during the 24 h ((**G**), unpaired *t* test, ** *p* < 0.01), 12 h light-on ((**H**), two-way ANOVA, **** *p* < 0.0001**)** and hourly time course during 24 h ((**I**), two-way ANOVA, main effect of genotype, F (1, 720) = 35.07; **** *p* < 0.0001, effect of genotype × time interaction, F (23, 720) = 3.073; **** *p* < 0.0001), but no significant changes in 12 h light-off ((**H**), two-way ANOVA, *p* > 0.05). (**J**–**L**): Mice spend more Wake time in 12 h light-on ((**K**), two-way ANOVA, * *p* < 0.05) and hourly time course during 24 h ((**L**), two-way ANOVA, main effect of genotype, F (1, 720) = 4.943; * *p* < 0.05, effect of genotype × time interaction, F (23, 720) = 1.596; * *p* < 0.05), but no significant increase in total 24 h ((**J**), unpaired *t*-tests, *p* >0.05) and 12 h light-off ((**K**), two-way ANOVA, *p* > 0.05). * Tukey’s post hoc comparison at time points in an hourly time course over 24 h, * *p* < 0.05. The black bar represents the period of light-off time in (**F**,**I**,**L**).

We recently reported that aging (12 mo) DPP6-KO mice have circadian dysfunction and might have sleeping issues, as determined by home-cage locomotor activity [19]. Compared to WT, aging DPP6-KO mice show an increase in hourly locomotor activity, and the circadian rhythm/clock is off at certain time points, especially during the light-on period [19]. It is unclear from activity measures, however, whether sleep is disrupted in these mice. To further investigate this phenotype and directly observe sleep patterns in this study, we measured sleep stages based on electroencephalogram (EEG), electromyogram (EMG), behavioral activity and body temperature data using the DSI implant wireless recording system (Figure 1A). EEG data showed that NREM sleep is dominated by high-amplitude and low-frequency activity; delta power is mostly present from medium to high levels, while theta is present from low to medium levels, and EMG shows low muscle tone and no locomotor activity. During REM sleep, also known as paradoxical sleep, EEG shows high-frequency, low-amplitude activity, and high theta power, but EMG is nearly absent and there is no locomotor activity. During the Wake stage, EEG is low-amplitude, and both delta and theta power levels are low, while the high EMG-neck muscle tone indicates high-amplitude locomotor activity (Figure 1B). The normal mouse sleep sequence is Wake–NREM–REM–Wake (Figure 1C).

To investigate DPP6’s role in regulating sleep, we first measured sleep in aging 12 mo WT and DPP6-KO mice. We recorded physiological measures of EEG/EMG/activity/body temperature and collected data for 2 days. Data from each day was binned across 12 h light-on (6 AM, sleep cycle) and light-off (6 PM, movement cycle) periods, as well as hourly over a 24 h time course. Furthermore, physiological data was used to categorize NREM, REM and Wake time periods. At the same time, we also measured locomotor activity and body temperature. Compared to the aging WT, we found that 12 mo DPP6-KO mice exhibited a significant decrease in NREM sleep during the 12 h light-on, but not in the 12 h light-off period (Figure 1E). NREM time was also significantly reduced across a 24 h time course in both light-on and off periods (Figure 1F), but there was no significant difference in the total 24 h NREM sleep time (Figure 1D).

REM sleep is important for various cognitive functions like memory consolidation [23] and is linked to Alzheimer’s disease, depression and dementia pathophysiology [24]. We found that REM sleep is significantly increased over the total 24 h in 12 mo DPP6-KO mice (Figure 1G), especially in 12 h light-on period and several of the hourly bins during the 24 h time course (Figure 1H,I) but not significantly changed in the 12 h light-off period (Figure 1H). Consistent with these results, 12 mo DPP6-KO mice also showed more Wake time in the 12 h light-on period (Figure 1K), and Wake time also significantly increased during the hourly time course (Figure 1L), although there were no differences in the total 24 h a day (Figure 1J) and the 12 h light-off period (Figure 1K).

**Figure 2:**
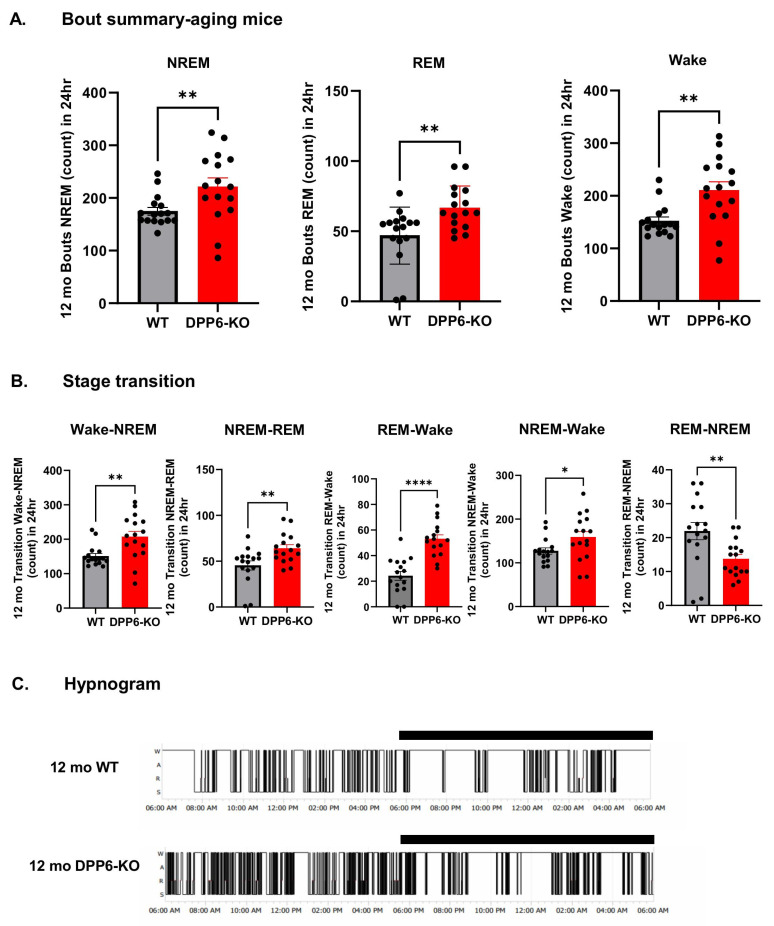
**Aging DPP6-KO mice have fragmented sleep with significant increase in bout counts and transition times.** (**A**): Aging DPP6-KO mice have increased bout summary counts compared to WT during 24 h in NREM (n = 8 mice each, ** p < 0.01), REM (** *p* < 0.01) and Wake (** *p* < 0.01). (**B**): Increased transition times in Wake–NREM (** *p* < 0.01), NREM-REM (** *p* < 0.01), REM–Wake (**** *p* < 0.0001), and NREM–Wake (* *p* < 0.05), and decreased in REM-NREM (** *p* < 0.01). All statistical analyses were under unpaired *t*-test or Mann–Whitney U test. (**C**): Example hypnogram sleep patterns during 24 h show that aging DPP6-KO mice have fragmented sleep. Black bar represents period of light-off time.

To determine sleep quality, we analyzed the bout summary counts and transition times from one sleep stage to the others that were generated by NeuroScore (v3.3) analysis (See the Methods Section). Increased bout summary and sleep transition times are primarily attributed to sleep disruptions in human aging and neurodegenerative diseases, especially in AD/dementia patients [25]. We found that bout summary counts of NREM and REM sleep and Wake stage are significantly increased in 12 mo DPP6-KO mice compared to WT during a 24 h day cycle from light-on to light-off (Figure 2A). Also, Wake–NREM, NREM-NEM, REM–Wake and NREM–Wake transition times significantly increased in 12 mo DPP6-KO mice during 24 h (Figure 2B), although the REM-NREM transition time decreased. We also found the same results from hypnogram sleep patterns of 12 mo DPP6-KO mice during 24 h (Figure 2C). It showed dense, fragmented hypnogram patterns that exhibited numerous shifts between sleep stages and awakenings; this indicates that 12 mo DPP6-KO mice have impaired sleep continuity with frequent awakenings and short, disrupted sleep. These results suggest that 12 mo DPP6-KO mice have sleep disorders with significant fragmentation, in which sleep is broken into smaller and discontinuous segments by interruptions, and the sleep architecture is not continuous and smooth. This shows that they not only have less-deep NREM sleep, but they also show fragmented sleep with REM rebound compared to 12 mo WT.

### 2.2. Three-Month-Old Adult DPP6-KO Mice Have Abnormal Sleep with Increased REM

**Figure 3:**
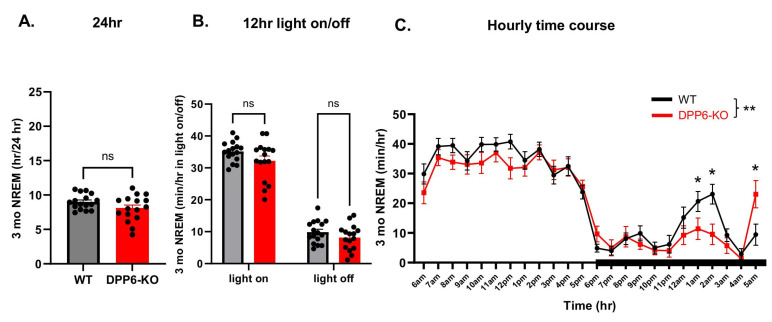

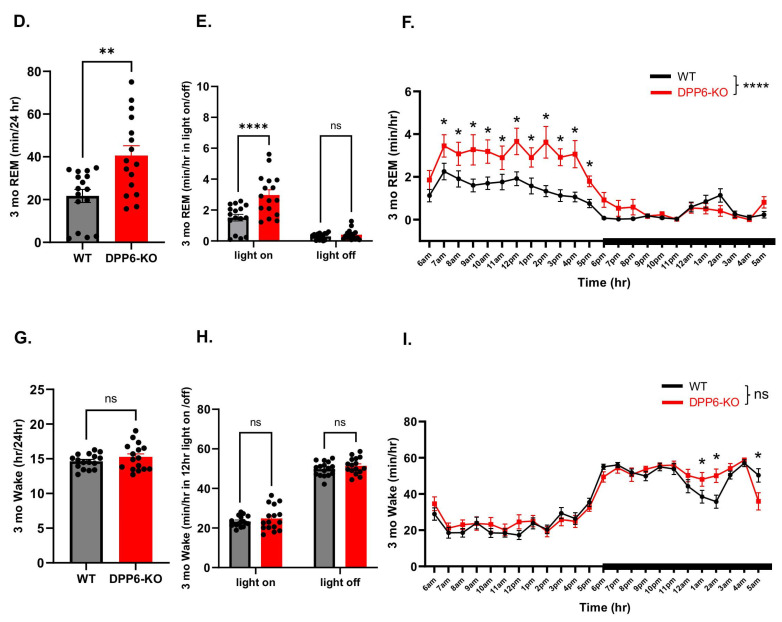
**Adult DPP6-KO mice have abnormal sleep with increased REM sleep.** (**A**–**C**): Adult DPP6-KO mice have no significant differences in total NREM sleep during 24 h ((**A**)**,** n = 8 mice each, unpaired *t*-test, *p* > 0.05) and 12 h light-on/off ((**B**), two-way ANOVA, *p* > 0.05), but a significant difference in hourly time course during 24 h ((**C**), two-way ANOVA, main effect of genotype: F (1, 720) = 8.188; ** *p* < 0.01, effect of genotype × time interaction: F (23, 720) = 1.770; * *p* < 0.05) compared to WT, with decreased NREM sleep time at 1 AM and 2 AM, increased at 5 AM (* Tukey’s post hoc comparison: * *p* < 0.05). (**D**–**F**): Increased total REM sleep during 24 h ((**D**), unpaired *t*-test, ** *p* < 0.01), 12 h light-on ((**E**), two-way ANOVA, **** *p* < 0.0001) and hourly time course during 24 h ((**F**), two-way ANOVA, main effect of genotype: F (1, 720) = 61.74; **** *p* < 0.0001, effect of genotype × time interaction: F (23, 720) = 2.785; **** *p* < 0.0001. * Tukey’s test post hoc comparison, *p* < 0.05), and no significant changes in 12 h light-on ((**E**), two-way ANOVA, *p* > 0.05). (**G**–**I**): No significant changes in Wake time in total 24 h ((**G**), unpaired *t*-test, *p* > 0.05) and 12 h light-on/off ((**H**)**,** two-way ANOVA, *p* > 0.05); in hourly time course during 24 h there is no effect of genotype, but there is an effect of the interaction between time and genotype ((**I**), two-way ANOVA, genotype effect: F (1, 720) = 3.252; *p* > 0.05, interaction effect: F (23, 720) = 1.759; * *p* < 0.05. * Tukey’s post hoc comparison, *p* < 0.05). Black bar represents period of light-off time in (**C**,**F**,**I**).

**Figure 4:**
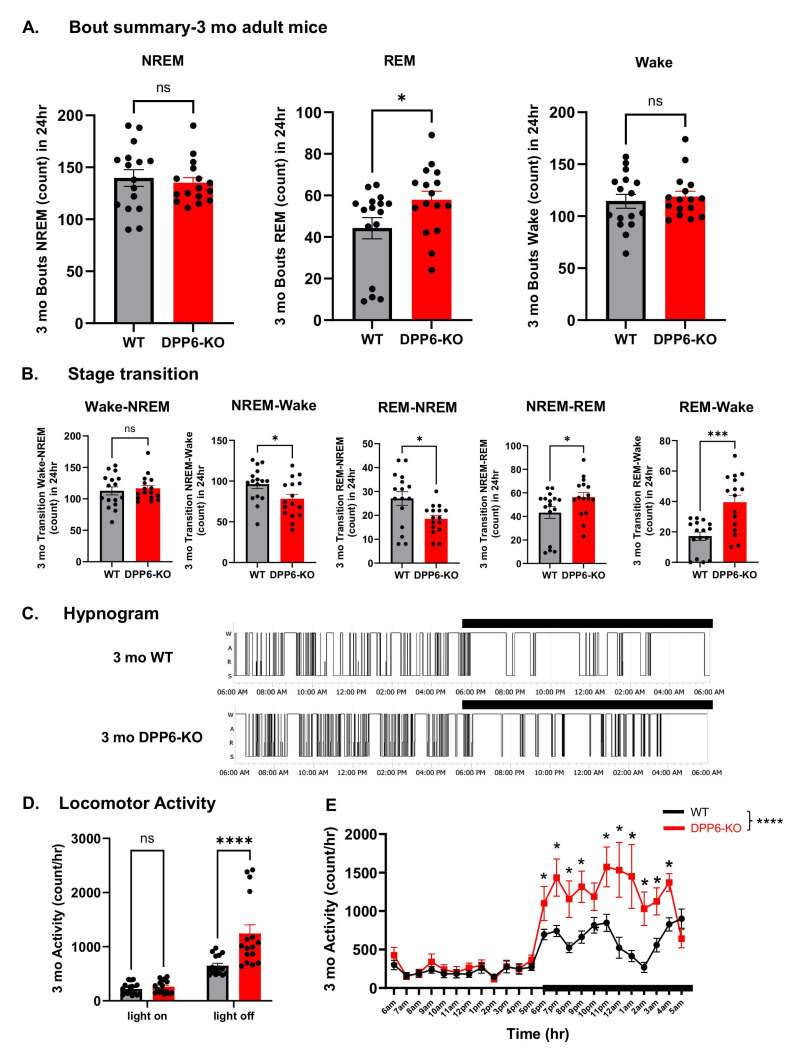
**Adult DPP6-KO mice have mildly fragmented sleep with normal bout summary counts in NREM and Wake.** (**A**): Adult DPP6-KO mice have no significant differences in bout summary counts compared to WT during 24 h in NREM (n = 8 mice each, *p* > 0.05) and Wake (*p* > 0.05), but a significant increase in REM (* *p* < 0.05). (**B**): No changes in transition times in Wake–NREM (*p* > 0.05), but a decrease in NREM–Wake (* *p* < 0.05) and REM-NREM (* *p* < 0.05), and an increase in NREM-REM (* *p* < 0.05) and REM–Wake (*** *p* < 0.001). All statistical analyses were under unpaired *t*-test. (**C**): Example hypnogram sleep patterns during 24 h show that adult DPP6-KO mice have fragmented sleep. Black bar represents period of light-off time. (**D**,**E**): Adult DPP6-KO mice show increased locomotor activity during 12 h light-off ((**D**), two-way ANOVA, **** *p* < 0.0001) and hourly time course during 24 h ((**E**), two-way ANOVA, main effect of genotype: F (1, 720) = 64.49; **** *p* < 0.0001, effect of genotype × time interaction: F (23, 720) = 3.620; *p* < 0.0001. * Tukey’s post hoc comparison, *p* < 0.01).

After observing that aging 12 mo DPP6-KO mice exhibit sleep disorders, we examined whether adult 3 mo DPP6-KO mice have the same dysregulation. We collected electrophysiological data (EEG/EMG/activity/body temperature) from 3 mo WT and DPP6-KO mice. Surprisingly, there were no significant differences between 3 mo DPP6-KO mice and WT in NREM sleep (Figure 3A,B) or Wake time (Figure 3G,H) across 24 h or 12 h light-on/off periods. However, we did find a significant difference in the hourly time course during the 24 h (Figure 3C,I). Post hoc comparisons revealed a decrease in NREM and an increase in Wake time at 1 AM and 2 AM in 3 mo DPP6-KO mice during light-off (Figure 3C,I), relative to WT controls, but there is no genotype effect in Wake time (Figure 3I). Like what we observed in 12 mo DPP6-KO mice, REM sleep was significantly increased in 3 mo DPP6-KO mice during a 24 h period (Figure 3D), the 12 h light-on period, and across the 24 h hourly time course (Figure 3E,F). We also analyzed the bout summary counts and transition times to determine the quality of sleep. There were no significant differences in bout summary counts for the NREM or Wake stages, but there was an increase in REM stage bouts during a 24 h day cycle in 3 mo DPP6-KO mice compared to WT (Figure 4A). Similarly, transition times were no different for Wake–NREM, but NREM–Wake and REM-NREM transition times were significantly decreased. Transitions related to REM (e.g., NREM-REM and REM–Wake) significantly increased due to the greater overall REM in 3 mo DPP6-KO mice throughout the light phase (Figure 4B). We also found similar results from hypnogram sleep patterns that showed slightly dense, fragmented and more REM shifts in 3 mo DPP6-KO mice during a 24 h day cycle (Figure 4C). Overall, 3 mo KO mice exhibited a prominent increase in REM sleep compared to WT. The increased REM may be needed for restorative sleep, to recover from the day’s physical and mental exertions, due to the increased locomotor activity [26]. This is supported by our finding that the locomotor activity was also significantly increased in movement cycles for both the 12 h light-off period (Figure 4D) and across the 24 h hourly time course (Figure 4E) in 3 mo DPP6-KO mice compared to WT. Taken together, these results suggest that adult DPP6-KO mice have mild insomnia and altered sleep architecture compared to adult WT mice.

### 2.3. Aging DPP6-KO Mice Display Depressive Behavior Related to Increased REM Sleep

**Figure 5:**
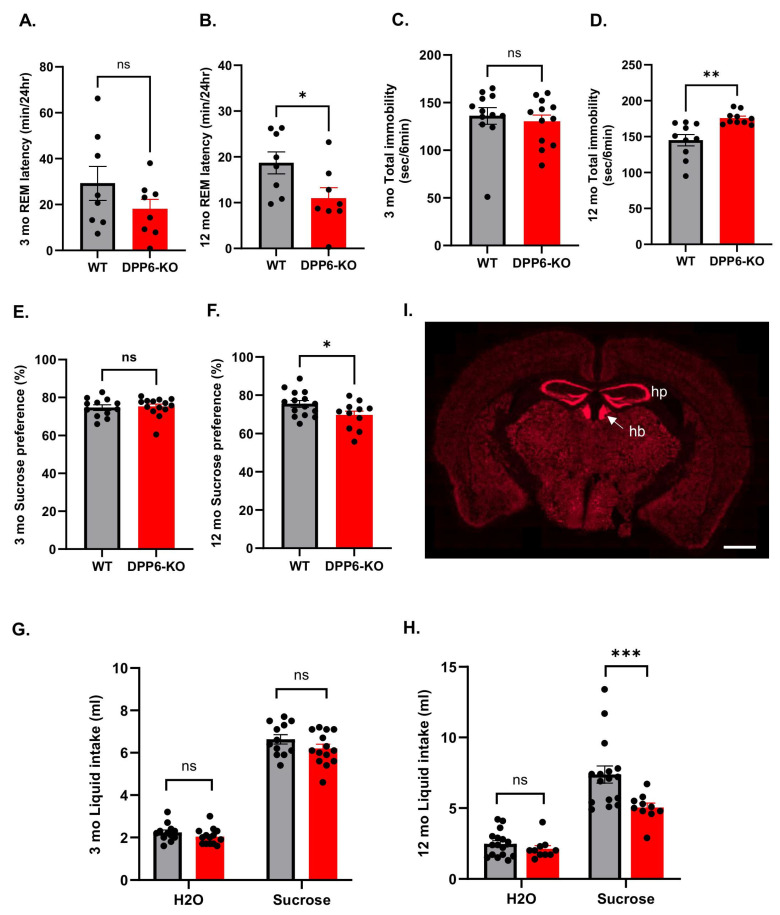
**Aging DPP6-KO mice exhibit depressive behavior.** (**A**,**B**): REM latency is not significantly different in adult DPP6-KO ((**A**), n = 8 each, *p* > 0.05), but significantly shorter in aging DPP6-KO, compared to WT ((**B**), n = 8 each, * *p* < 0.05). (**C**,**D**): Tail suspension test shows that immobility is significantly increased in aging DPP6-KO ((**D**), n = 10 each, ** *p* < 0.01) compared to WT; however, there are no significant changes in adult ((**C**), n = 12 each, *p* > 0.05) compared to WT. (**E**–**H**): Sucrose preference task shows that there is no significant changes in preference in adult DPP6-KO ((**E**), WT, n = 12, DPP6-KO, n = 14, *p* > 0.05) and intake of 1% sucrose solution ((**G**), two-way ANOVA, *p* > 0.05), but there is a significant decrease in preference in aging DPP6-KO ((**F**), WT, n = 15, DPP6-KO, n = 11,* *p* < 0.05) and intake of 1% sucrose solution ((**H**), two-way ANOVA, *** *p* < 0.001), compared to WT. (**I**): mRNA level of DPP6 (red) strongly expressed in hippocampus and habenula in WT brain. hp, hippocampus; hb, habenula. Scale bar: 1 mm. All statistical analyses were under unpaired *t*-test in (**A**–**F**).

We found that aging DPP6-KO mice show increased REM sleep (Figure 1G–I), which is a biomarker and common finding in patients with late-life depression and can coexist in AD patients, though AD itself is typically associated with decreased REM sleep. Patients with depression often show a shortened REM latency, which is the time it takes to enter REM sleep after falling asleep, as well as increased REM sleep duration [27,28,29]. Given the prevalence of depression in patients with AD, we wanted to further investigate symptoms associated with depression in DPP6-KO mice. First, we analyzed the REM latency from EEG data with both 3 mo and 12 mo DPP6-KO mice and the WT control. We found that there is no significant decrease in REM latency in the 3 mo DPP6-KO compared to WT (Figure 5A), but 12 mo DPP6-KO mice show a significant decrease in REM latency compared to 12 mo WT (Figure 5B). To examine whether DPP6-KO mice have depression-like behaviors that align with observations of increased REM sleep, we performed two main depression-like behavioral tasks in both 3 mo and 12 mo mice. First, we performed the tail suspension test, in which we measured the total time of immobility. The results showed that there is a significant increase in immobility in 12 mo but not 3 mo DPP6-KO mice compared to WT (Figure 5C,D), implying more-severe depressive states in aging DPP6-KO mice. We then performed the sucrose preference task with the two-bottle choice to assess anhedonic behavior, a common symptom of depression in humans. We found that 12 mo DPP6-KO exhibit decreased intake of and preference for 1% sucrose solution, indicating a reduced ability to experience pleasure compared to WT (Figure 5F,H). However, no significant difference in sucrose intake and preference for 1% sucrose was observed in 3 mo DPP6-KO mice (Figure 5E,G). Notably, we also detected the mRNA level of DPP6 by RNAscope in adult WT mouse brain sections (Figure 5I), and found that mRNA of DPP6 is highly expressed in the hippocampus and habenula; the latter nucleus is involved in depression, emotional stability and anxiety, and has been linked to REM sleep [30,31,32]. Overall, these results suggest that adult 3 mo DPP6-KO mice exhibiting increased REM sleep needed recovery from the intense daily activity and fragmentation of sleep. In contrast, REM changes from adults to aging 12 mo DPP6-KO mice may be symptoms associated with both AD and depression, likely due to neurodegeneration in both the hippocampus and habenula.

### 2.4. DPP6 Increases NREM Sleep Time and Quality, and Improves Fragmented Sleep in 12 mo WT Mice with In Vivo Overexpressed DPP6

**Figure 6:**
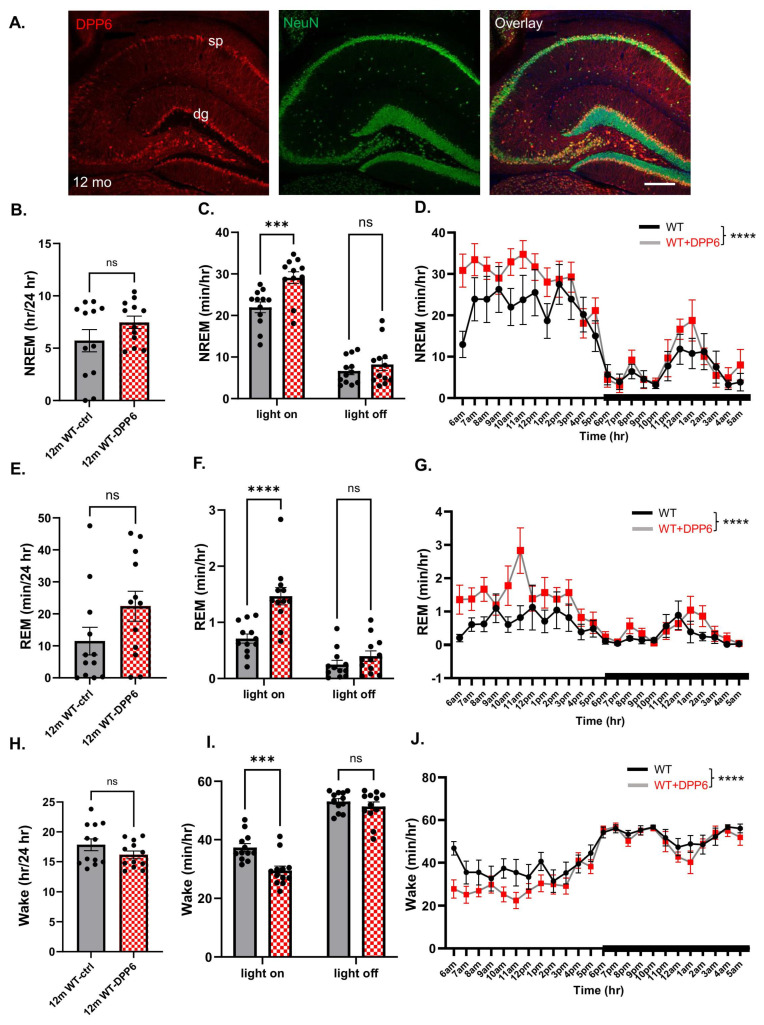
**DPP6 overexpression enhances the time spent in NREM and decreases wakefulness.** (**A**): Immunofluorescence labeling with NeuN (green, 1:1000) and BioID (red, 1:6000) antibodies confirms that DPP6 expression in brain section lasts for 12 months, scale bar: 100 µm. (**B**–**D**): When DPP6 is overexpressed in aging WT for year, NREM is significantly increased in 12 h light-on ((**C**), n = 6 mice each, two-way ANOVA, *** *p* < 0.001) and hourly time course during 24 h with main effect of genotype ((**D**), two-way ANOVA, main effect of genotype: F (1, 528) = 17.20; **** *p* < 0.0001, interaction effect: F (23, 528) = 0.970; *p* > 0.05) compared to control WT with AAV–control, in which there is no significant increase in total 24 h ((**B**), unpaired *t*-test, *p* > 0.05) and 12 h light-off ((**D**), two-way ANOVA, *p* > 0.05). (**E**–**G**): Increased REM in 12 h light-on ((**F**), two-way ANOVA, **** *p* < 0.0001) and hourly time course during 24 h with main effect of genotype ((**G**), two-way ANOVA, main effect of genotype: F (1, 528) = 24.51; **** *p* < 0.0001, interaction effect: F (23, 528) = 1.375; *p* > 0.05) compared to WT-AAV–control, but there are no changes in 12 h light-off ((**F**), two-way ANOVA, *p* > 0.05) and no significant increase in total 24 h ((**E**), unpaired *t*-test, *p* < 0.05). (**H**–**J**): Decreased Wake time in 12 h light-on ((**I**), two-way ANOVA, *** *p* < 0.001) and hourly time course during 24 h ((**J**), two-way ANOVA, main effect of genotype: F (1, 528) = 19.28; **** *p* < 0.0001, interaction effect: F (23, 528) = 1.028; *p* > 0.05.), but no significant changes in total 24 h ((**H**), unpaired *t*-test, *p* > 0.05) and 12 h light-off ((**I**), two-way ANOVA, *p* > 0.05).

**Figure 7:**
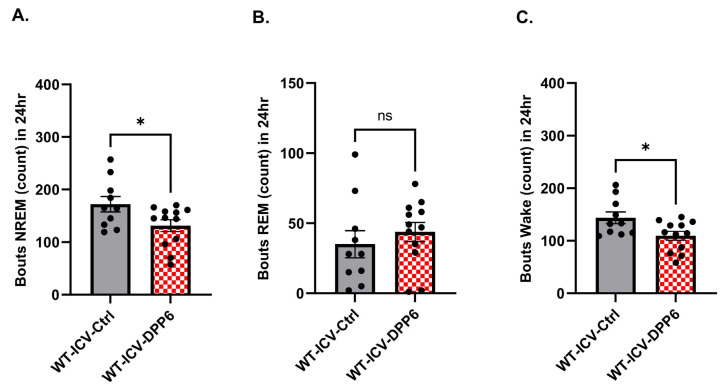
**DPP6 improves sleep quality with less disturbed sleep.** (**A**): The overexpressed-DPP6 aging WT shows a greater decrease in NREM bout counts (* *p <* 0.05) compared to the AAV–control. (**B**): Bout summary counts in REM have no significant differences in the overexpressed-DPP6 aging WT (*p* > 0.05) compared to the AAV–control. (**C**): Aging WT with overexpressed DPP6 shows a significant decrease in bout count in Wake (* *p* < 0.05) compared to AAV–control. All statistical analyses were under unpaired *t*-test.

Since we found significant dysregulation of sleep in aging DPP6-KO mice, we wanted to further probe DPP6’s role in the regulation of sleep. To do this, we overexpressed DPP6 to examine whether sleep disorders can be improved in aging mice. We performed ICV injection with AAV-DPP6 or AAV–control at P0–P2 of WT pups and waited 12 mo for expression of DPP6. Immunofluorescence was conducted with NeuN and DPP6 antibodies to confirm that DPP6 is expressed in the brain sections of injected mice after 12 mo (Figure 6A). DPP6-overexpressing mice at 12 mo were implanted with electrodes and we collected EEG/EMG/activity/body temperature data for two days. We found that the NREM sleep is significantly increased and the wake time is significantly decreased during the 12 h light-on sleep cycle in the DPP6-overexpressing mice compared to the control mice (Figure 6C,I). Also, there is a significant increase in NREM and a decrease in Wake time during the 24 h hourly time courses (Figure 6D,J), while there are no significant differences in the NREM and Wake stages in the total 24 h day cycle (Figure 6B,H). We also found that REM sleep is still increased during the 12 h light-on, but not in light-off (Figure 6F), and there is a significant increase in REM during the 24 h hourly time courses (Figure 6G), while there is no significant increase in REM during the 24 h day cycle. The bout summary counts showed that there is a significant decrease in NREM (Figure 7A) and in the Wake stage (Figure 7C), and no significant differences in counts of REM bouts during the 24 h day cycle (Figure 7B). Study of the transition times of stages showed that there are no changes in NREM-REM, REM–Wake, or in REM-NREM times, but there is a significantly reduced transition time from NREM–Wake, indicating that the mice have more stable sleep with less Wake time in the DPP6-overexpressed mice compared to the control mice.

We then compared the bout summary counts between the WT and DPP6-KO in the 3 mo adult versus the 12 mo aging mice and compared these to the 12 mo WT with overexpressed DPP6. During development, from adults at 3 mo to aging 12 mo mice, the bout count of NREM changed from no difference in adult 3 mo (Figure 4A) to significantly increased in aging 12 mo DPP6-KO (Figure 2A); but WT mice with overexpressed DPP6 showed a significant decrease in the bout count of NREM compared to the control group (Figure 7A). The bout count of REM increased in both adult 3 mo (Figure 4A) and aging 12 mo DPP6-KO mice (Figure 2A); in contrast, there were no significant changes in the bout count of REM in the WT with overexpressed DPP6 (Figure 7B). The bout count of the Wake stage changed from no difference in 3 mo (Figure 4A) to a significant increase in 12 mo DPP6-KO mice (Figure 2A); in contrast, there was a significant reduction in the bout Wake count in overexpressed DPP6 WT compared to the control group (Figure 7C). Similar results were seen from transitions in Wake–NREM, NREM-REM and REM–Wake. Overall, these data suggest that DPP6-KO mice start exhibiting mild sleep problems as adults (~3 mo) and this dysregulation was more pronounced at later ages (~12 mo). Sleep disorders in aging DPP6-KO mice are characterized by increased bouts in each sleep stage that indicate fragmented sleep, while overexpression of DPP6 in WT mice seems to promote sleep stability and time by reducing sleep bout counts and increasing the NREM time. Altogether, these results suggest that DPP6 contributes to the regulation of sleep by promoting sleep time and quality.

### 2.5. DPP6 Affects Body Temperature During Development

Body temperature is associated with metabolism, and DPP6 affects metabolism; as we have published, the body weight of DPP6-KO mice is lower from 1 month after birth and remains lower throughout their adult life to 12 months compared to WT at the same ages [19,33]. DPP6 mRNA expression is high in human pancreatic islets, where it is found in both insulin-producing beta cells and glucagon-producing alpha cells. Lipid mass spectrometry analysis indicates that DPP6 could be associated with lipid metabolism regulation. Changes in lipid metabolism can lead to cell death, which may affect muscle tissue, and muscle activity affects body temperature [34,35].

**Figure 8:**
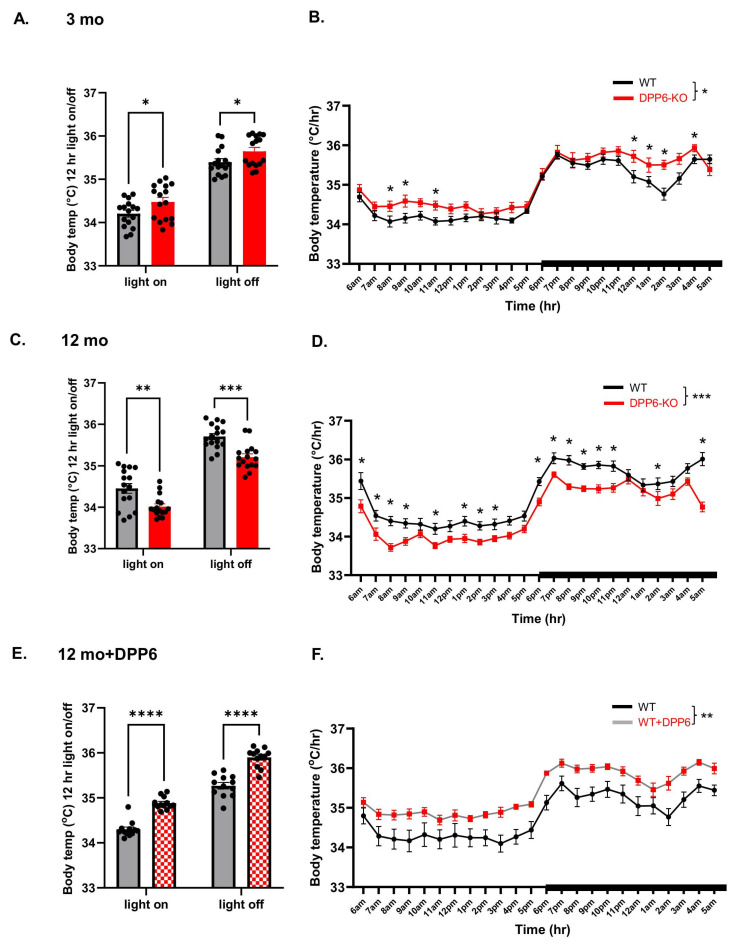
**Aging DPP6-KO mice show lower body temperature.** (**A**,**B**): Adult DPP6-KO mice have slightly higher body temperature compared to WT in 12 h light-on/off ((**A**), n = 8 mice each, two-way ANOVA, * *p* < 0.05) and hourly time course during 24 h ((**B**), two-way ANOVA, main effect of genotype effect: F (1, 30) = 4.758; * *p* < 0.05, interaction between genotype and time on body temperature, F (23, 690) = 2.269; *p* < 0.001. * Tukey’s post hoc comparison, *p* < 0.05). (**C**,**D**): Aging DPP6-KO mice have a significant decrease in body temperature compared to WT in both 12 h light-on/off ((**C**), n = 8 mice each, two-way ANOVA, ** *p* < 0.01 in light-on and *** *p* < 0.001 in light-off) and hourly time course during 24 h ((**D**), two-way ANOVA, main effect of genotype effect: F (1, 30) = 18.13; *** *p* < 0.001, interaction effect: F (23, 690) = 2.640; *p* < 0.0001. * Tukey’s post hoc comparison, *p* < 0.05). (**E**,**F**): Aging WT mice with overexpressed DPP6 show a significant increase in body temperature compared to WT-AAV–control in both 12 h light-on/off ((**E**), n = 6 mice each, two-way ANOVA, **** *p* < 0.0001), and hourly time course in 24 h showed main effect of genotype, no effect on interaction ((**F**), two-way ANOVA, genotype effect: F (1, 22) = 9.806; ** *p* < 0.01, interaction effect: F (23, 506) = 0.6278; *p* > 0.05).

Here, we collected data on body temperature during EEG detection. We found a significant increase in body temperature in the 12 h light-on/off periods and also during hourly time courses across 24 h, in 3 mo DPP6-KO mice compared to WT (Figure 8A,B). In contrast, 12 mo DPP6-KO mice had lower body temperatures in general, especially in both light-on/off compared to WT, as well as during the hourly time courses over 24 h (Figure 8C,D). This might be caused by lower metabolism in aging DPP6-KO mice. Surprisingly, 12 mo WT mice with overexpressed DPP6 showed increased body temperature in both the light-on/off cycle and in the hourly time courses over 24 h as compared to the control mice (Figure 8E,F). Overall, 3 mo DPP6-KO have ~0.3 °C increase in body temperature, while the 12 mo ones have a ~0.5 °C decrease; thus, from adult to aging, the DPP6-KO body temperature dramatically changes, likely due to increased activity in adults and lower metabolism in aging. These data suggest that DPP6 increases the metabolic rate in aging mice and may play a role in metabolism that affects body temperature.

## 3. Discussion

### 3.1. Sleep Dysfunction in DPP6-KO Mice Parallels Alzheimer’s Disease Progression

During the last decade, DPP6 has been reported to link to several neurodegenerative diseases [36,37,38,39,40,41,42,43,44]. Recently, DPP6 has been reported to be associated with Alzheimer’s disease (AD). Cacace et al. found significantly higher rare variants of DPP6 in early-onset AD and frontotemporal dementia, and loss of DPP6 expression and function had a significant impact on dementia [45], although the association of DPP6 and dementia is not clear in all cases [46,47]. In our group, we found that DPP6 plays a direct role in AD; aging DPP6-KO mice show symptoms of enhanced AD/dementia associated with a novel structure, i.e., clusters of large puncta that are abnormal presynaptic swellings, associated with synapse loss and neuronal death [18,19,48]. A new report showed that DPP6 is one of the RNA-edited genes involved in APOE-specific alteration in human AD brains [49]. DPP6 has also been linked to several neurodevelopmental disorders, including ASD [48,50]. Other recent reports, including a genome-wide association study, showed that DPP6 is a risk gene of cognitive decline in Parkinson’s disease [51,52]. Here, we add to this evidence by showing that DPP6 appears to also regulate sleep, metabolism, and symptoms of depression in mice.

### 3.2. Depression-like Symptoms in Aging DPP6-KO Mice Linked to REM Sleep

Our results also show that DPP6-KO mice exhibit depression-like symptoms in two behavioral tasks: tail suspension and sucrose preference. Depression is strongly linked to AD and can be both a risk factor for AD and a symptom of the disease itself; i.e., AD can cause depression. Thus, one of the most prevalent risk factors for AD is depression, affecting one million individuals, ~15%, out of 7 million AD/dementia patients. Epidemiological studies have also shown that a history of depression disorder across the lifespan significantly increases the risk of AD/dementia; chronic depression can damage the brain and thus increase the risk of AD. Depression may contribute to brain pathology, such as the accumulation of amyloid plaques, which are a hallmark of AD [53,54,55]. Therefore, preventing or reducing depression in the general population may reduce the risk of development of AD/dementia and, thus, significantly decrease the incidence of AD/dementia in older ages [56,57,58].

Increased REM sleep with shortened REM latency is also a biomarker and risk factor for depression [27,28,29]. Thus, increased REM sleep is linked to a higher likelihood of depressive symptoms, and REM sleep deprivation can temporarily alleviate depressive symptoms—a mechanism of some antidepressant drugs. This complex relationship is attributed to dysregulation in neurochemical pathways, particularly monoamines, and also involves the lateral habenula, which controls REM sleep [30]. In this study, we found that aging DPP6-KO mice have increased REM but also have shortened REM latency, which is not seen in AD patients who do not show signs of depression. In contrast, AD patients with concurrent depression symptoms have shortened REM latency. Similarly, aging DPP6-KO mice have depression disorders that we confirmed using the two main depression behavioral tasks. This suggests that aging DPP6-KO mice have both depression and AD, involving overlapping forms of neurodegeneration. Interestingly, DPP6 is highly expressed in the habenula, an area with a significant role in mood regulation [59]. The hyperactivity of the lateral habenula is linked to suppressing dopamine and serotonin, and this process is implicated in mood disorders like depression [60,61]. DPP6 may then play a role in regulating the excitability of habenular neurons.

### 3.3. DPP6 Regulates Metabolism and Thermoregulation in Aging

In our previous study, we found that DPP6-KO mice have significantly lower body weight (8–15% less) compared to WT. The body weight of DPP6-KO mice is lower from 1 month after birth and remains lower throughout their adult life to 12 months, compared to WT at the same ages. Having a normal body weight at P0 but decreasing body weight as the DPP6-KO mice age suggests a metabolic phenotype developing after birth [19,33]. Another study has reported a metabolic link between DPP6 and the glucose–insulin pathway [62]. Also, a clinical case report has revealed a DPP6-associated autoimmunity syndrome with systemic symptoms of gastrointestinal disturbance and weight loss, as well as sleep disturbances with cognitive dysfunction, seizures, weight loss, anxiety and depression [17,21]. In the present study, we found that DPP6-KO mice have significant body temperature changes from adult to aging compared to the same ages of WT controls. Thus, adult KO mice have increased body temperature apparently due to the higher activity, and aging KO mice show decreased body temperature associated with the lower metabolism that occurs during aging. We do not see similar changes in WT from adult to aging, suggesting that DPP6-KO mice show faster aging and more degenerative changes with aging than WT. This decrease in body temperature in DPP6-KO mice from adult to aging suggests that DPP6 may play a role in body temperature regulation across development.

### 3.4. Future Study Directions

Parvalbumin (PV) interneurons are fast-spiking GABAergic neurons essential for coordinating synchronous neuronal firing, which underlies proper emotional and cognitive function. These interneurons play critical roles in regulating cortical activity, sleep architecture, and memory consolidation [63]. In the context of sleep regulation, PV neurons are particularly active during REM sleep, where they suppress cortical activity and help maintain the brain’s excitation/inhibition balance. Dysfunction or loss of PV interneurons can disrupt this balance, leading to sleep instability and fragmentation [64].

PV interneuron dysfunction is also implicated in mood disorders. Chronic stress can alter PV interneuron phenotype and function, leading to pathological changes in circuits associated with depression and anxiety [65]. Reduced PV interneuron expression or activity has been linked to depressive behaviors and impaired emotional regulation [66]. Given that aging DPP6-KO mice exhibit both fragmented sleep and depressive-like behavior, examining whether PV interneuron function is compromised in these animals represents a promising avenue for future investigation. Such studies could elucidate whether DPP6-mediated effects on sleep and mood disorders involve alterations in PV interneuron activity or circuit integration.

Beyond sleep regulation, our findings regarding altered body temperature in aging DPP6-KO mice point to a broader role for DPP6 in metabolic homeostasis. Future studies will investigate potential mechanistic links between DPP6, metabolism, body weight regulation, and thermoregulation. Understanding these metabolic functions is particularly relevant given the well-established connections between metabolic dysfunction, sleep disturbances, and neurodegenerative diseases.

A particularly intriguing avenue for investigation is the potential interaction between DPP6 and Apolipoprotein E (APOE), the strongest and most prevalent genetic risk factor for late-onset Alzheimer’s disease. The ε4 allele of APOE affects more than half of AD patients and influences multiple pathological processes, including amyloid-beta (Aβ) accumulation, tau pathology, neuroinflammation, and lipid metabolism [67,68,69,70]. Given that APOE plays central roles in both brain lipid trafficking and systemic metabolism, and that DPP6 deficiency leads to age-dependent metabolic dysregulation, we hypothesize that DPP6 may modulate APOE function or expression. Such an interaction could provide a link between the metabolic, sleep, and mood disturbances observed in aging DPP6-KO mice. To identify broader metabolic pathways regulated by DPP6, we will investigate its potential interactions with proteins involved in lipid metabolism, mitochondrial function, energy homeostasis, and APOE-regulated pathways. Elucidating these molecular connections may reveal how DPP6 integrates metabolic signaling with neuronal function and could identify novel therapeutic targets for age-related sleep disorders, metabolic dysfunction, and neurodegenerative diseases.

## 4. Materials and Methods

### 4.1. Animals

We used 3-month-old (3 mo) adults and 12-month-old (12 mo) aging male mice of WT and DPP6-KO in this study. The mice were housed in the NINDS (National Institute of Neurological Disorders and Stroke) animal facility. All animal care and experimental procedures (Tail suspension task were performed under Dr. Cameron’s lab ASP) were approved by the National Institute of Child Health and Human Development Animal Care and Use Committee in accordance with NIH guidelines. Mice were weaned at 3 weeks of age, housed 3–4 mice per cage, and double-checked by genotyping. WT and DPP6-KO mice have the same background C5BL/6. Mice were housed under a 12 h light/dark cycle with the lights on at 6 AM and lights off at 6 PM. Animals with telemetries were housed individually.

### 4.2. Surgical Implantation

Implant surgery training was received from DSI (Harvard Bioscience, Minneapolis, MN, USA) in-person and based on their surgical manual and Lundt et al., 2016 [71]. Briefly, mice were implanted with HD-X02 wireless telemetry devices (DSI) with 2 electrodes for electroencephalogram (EEG) and 2 electrodes for electromyogram (EMG) under sterile techniques and isoflurane anesthesia (3% for induction and 1.5% for maintenance). EEG electrodes were inserted through the skull to contact the dura mater by using two stainless steel screws (DSI): one as negative (−) lead placed 1 mm anterior to the bregma and 1 mm lateral to the sagittal suture; the other screw as positive (+) lead was placed 3 mm posterior to the bregma and 3 mm contralateral to the sagittal suture. The EEG electrodes were fixed to the skull with dental acrylic integrity material. The two EMG electrodes were placed into neck muscles. Mice were individually housed in an animal cubicle room after surgery. For post-surgical analgesia, mice were provided with a Bio-serv carprofen tablet per day (Bio-serv, Flemington, NJ, USA) the day before and the following 3 days after the surgery. The mice recovered 10–14 days before we started to collect data.

### 4.3. In Vivo EEG/EMG/Body Temperature/Locomotion Recording

Surgically implanted mice were allowed to recover for 10–14 days after surgery. Animals were housed individually in standard plexiglass home cages and had receivers that transmit data from the EEG and EMG implants to a computer via the data exchange matrix using Ponemah software (Version 5.6x) (DSI). To determine the stages of sleep, body temperature and locomotion, we detected and recorded EEG/EMG/body temperature/locomotion on mice from 2- to 3-month-old adults and 12-month-old aging WT and DPP6-KO mice under a 12 h light/dark cycle with the lights on at 6 AM and lights off at 6 PM. Electrophysiological data were collected for 2 days. All recordings took place in the animal cubicle room with minimal noise or other background disturbances. The data were analyzed using NeuroScore software (DSI). The automatically defined sleep stages and Wake stage were recorded and then checked visually and corrected manually if necessary. The results were examined for a total of 24 h, 12 h light-on and off, and hourly time courses over the 24 h. Timeline summarizing the chronological sequence of procedures: Surgically implanted mice at 3 mo or 12 mo → Recovery for 10–14 days from surgery → started EEG recording for 2 days under 12 h light/dark cycle → collected data → analysis of EEG/EMG, locomotion activity and body temperature.

### 4.4. EEG Data Analysis

Sleep scoring was conducted using NeuroScore Software v3.3 from DSI. Raw telemetry data, including EEG, EMG, locomotor activity and body temperature readings, were imported into NeuroScore from Ponemah. These signals were then bandpass-filtered (EEG at 0.5–100 Hz and EMG at 10–100 Hz) and assessed in 10 s intervals. Stages were classified as Wake, rapid eye movement (REM) sleep, or non-REM (NREM) sleep, utilizing the NeuroScore Mouse Sleep scoring module. Frequencies within the delta band ranged from 0.5 to 4 Hz, and the theta band frequencies used for scoring ranged from 6 to 9 Hz. Wakefulness was characterized by a mix of variable high-frequency EEG signals alongside increased EMG activity. NREM sleep was defined by low-frequency, high-amplitude EEG signals and reduced EMG activity. In contrast, REM sleep was characterized by a predominance of theta frequencies in the EEG (with a theta/delta ratio greater than 3) and decreased EMG activity. Sleep was scored in 10 s intervals. At least two successive 10 s intervals of the same sleep stage defined a continuous sleep segment. NeuroScore can generate counts of identified sleep bouts and transition times from one sleep stage to another for the entire recording period by providing features that include identification and counting of different sleep stages. These specific counts of sleep bouts are used to understand the fragmentation of sleep, which helps in diagnosing and assessing the severity of various sleep disorders, particularly sleep apnea and insomnia [72]. Regarding telemetry locomotion activity data, the maximum activity count per 10 s epoch was determined, and the cumulative activity counts per hour and the total activity within 12–24 h were derived.

### 4.5. Neonatal Intracerebroventricular (ICV) Injection

Neonatal ICV injection was performed following the protocol from the NICHD Microscopy and Imaging Core, based on the method in Kim [73]. Briefly, P0–P2 neonates were anesthetized with hypothermia on ice. A 10 mL Hamilton syringe with a 32-gauge needle was used for injection. Depending on the titer, 1–5 μL total of recombinant adeno-associated virus (AAV9.hSyn.hGHintron-control or DPP6 with titer 4–5 × 10^10^/μL) was manually injected slowly into the brain’s lateral ventricles, bilaterally. Injections were administered at a depth of 2.5 mm, with the injection sites at 1/3 of the distance from the lambda suture to each eye, approximately 1–1.5 mm lateral to midline. After injection, the needle was held at the injection site for 10 s and then slowly withdrawn. Then, the pup was placed back on the warming pad until its body temperature and skin color returned to normal and the pup started to move. The pups were then returned to their biological mother after they recovered normal movement. The cage was monitored for several minutes to ensure the dam accepted all pups. Following injections, pups were monitored for 2 weeks.

### 4.6. RNAscope Assay

We performed the assay in fixed-frozen mouse brain sections by using RNAscope^®^ Multiplex Fluorescent v2 Assay kit (Cat# 323100, ACD, Newark, CA, USA). We completely followed the manufacturer’s instructions (https://acdbio.com/rnascope-multiplex-fluorescent-v2-assay) (accessed on 15 October 2024). Briefly, RNA detection uses a proprietary probe hybridization and amplification system. Fixed-frozen 8 μm mouse brain sections were post-fixed in 4% PFA diluted in PBS for 15 min at room temperature, then dehydrated in EtOH (50, 70, 100%) and rehydrated in hydrogen peroxide for 10 min; then target retrieval was performed for 5 min at 99 °C. The protocol of RNA detection followed the manufacturer’s instructions, with probe hybridization by incubating the sample with the probe of DPP6 transcript variant 1 mRNA (Cat# 492411-C3, ACD) and amplification by its amplifier to create a signal that can be visualized by a fluorescent dye. Mounting the sections was performed with ProLong Diamond Antifade Mountant with DAPI to visualize the neuron nuclei. We imaged the slides using either a Zeiss 710 microscope (Zeiss, Oberkochen, Germany) or a Carl Zeiss AxioScan Z1 slide scanner (Zeiss, Oberkochen, Germany).

### 4.7. Immunofluorescence in Mouse Brain Sections

We used the methods in Lin et al. 2022 [19]. In brief, mice were perfused with 4% paraformaldehyde (PFA) in PBS. The collected brains were placed in 4% PFA and then equilibrated in 30% sucrose for 24–48 h. A series of equidistant frozen 8 μm coronal sections were prepared. The sections were taken from the Bregma range −1.955 to −2.095 mm. The sections were permeabilized and blocked with buffer (0.3% TritonX-100 + 1% normal goat serum in 1× PBS), Then, sections were incubated overnight with the primary chicken antibody for BioID2 (BID2-CP-100 at 1:6000 dilution, BioFront technologies, Tallahassee, FL, USA) to label DPP6 and rabbit antibody for NeuN (ABN78 at 1:500 dilution, Millipore Sigma, Burlington, MA, USA) at 4 °C, followed by the fluorescent probe-conjugated secondary antibodies (Thermo Fisher, Waltham, MA, USA. 1:800 dilution) for 2 h at RT. Nuclei were counterstained with DAPI. Immunofluorescence (IF)-labeled slides were imaged either using a Zeiss 710 microscope or scanning the entire brain section or hippocampus using a Carl Zeiss AxioScan Z1 slide scanner (Zeiss, Oberkochen, Germany)with a 20× objective (Plan-Apochromat, NA 0.8) and a Colibri7 LED illumination source (Zeiss, Oberkochen, Germany).

### 4.8. Tail Suspension Test

The tail suspension test is a test to measure indices of “learned helplessness.” It was performed to evaluate depression-like behavior in mice [74]. Briefly, the mouse’s tail was secured with tape, and the mouse was suspended in a straight line with its body hanging freely. The duration of immobility was recorded over a 6 min test period by Open Broadcaster Software (OBS, version 31.0). We measured the total immobility time during the 6 min recording. A mouse exhibits alternating periods of agitation and immobility, referred to as the searching and waiting phases, respectively. The searching phase is characterized by body jerks, running motions and body torsions as the mouse attempts to catch its tail. Immobility is the waiting phase marked by the absence of initiated movements.

### 4.9. Sucrose Preference Test (Two-Bottle Choice)

Mice are given a choice between water and a sucrose solution. A reduced preference for the sucrose solution is interpreted as a sign of depression-like behavior. Bottle contents were weighed individually at the beginning and end to determine how much liquid had been consumed from each bottle. We followed a standard protocol [75]. Briefly, mice were individually housed to habituate drinking water with a feeder that can hold two bottles for 1–2 days. On the testing day, one bottle was replaced with 1% sucrose, and then both bottles were weighed. 12 h later, we switched the position of the two bottles to avoid the development of a side preference. At the 24 h time point, the bottles were weighed for the second time. Sucrose preference was calculated as a percentage of sucrose solution consumption in reference to the total liquid consumption: amount of sucrose solution consumed (1st weight of sucrose − 2nd weight of sucrose) × 100/total liquid consumption %.

### 4.10. Statistical Analysis

We used an unpaired *t*-test or two-way ANOVA for statistical analyses by GraphPad Prism 10.6.1 with selected normality (Shapiro–Wilk) and homogeneity of variance tests as default settings for all data analyses. Parametric or non-parametric statistical tests were performed accordingly. The n values and details of controls and comparisons used for statistical analyses are described for each experiment in the corresponding figure legends or within the Results Section. Two-way ANOVA provides the main effect of genotype or the interaction effect between genotype and time, with Tukey’s post hoc multiple comparisons used if there is an effect. All results are presented as the mean ± SEM. * *p*  <  0.05; ** *p*  <  0.01; *** *p*  <  0.001; **** *p*  <  0.0001; ns, non-significant (*p* > 0.05).

## Data Availability

Upon acceptance, the data underlying the study will be deposited in a cloud-based communal repository.
